# Random Matrix Transformation and Its Application in Image Hiding

**DOI:** 10.3390/s23021017

**Published:** 2023-01-16

**Authors:** Jijun Wang, Fun Soo Tan, Yi Yuan

**Affiliations:** 1 Faculty of Computing and Informatics, Universiti Malaysia Sabah (UMS), Kota Kinabalu 88400, Malaysia; 2 Guangxi Key Laboratory of Big Data in Finance and Economics, Nanning 530003, China; 3 Faculty of Big Data and Artificial Intelligence, Guangxi University of Finance and Economics, Nanning 530003, China

**Keywords:** random matrix, image hiding, image encoding, hybrid transmission

## Abstract

Image coding technology has become an indispensable technology in the field of modern information. With the vigorous development of the big data era, information security has received more attention. Image steganography is an important method of image encoding and hiding, and how to protect information security with this technology is worth studying. Using a basis of mathematical modeling, this paper makes innovations not only in improving the theoretical system of kernel function but also in constructing a random matrix to establish an information-hiding scheme. By using the random matrix as the reference matrix for secret-information steganography, due to the characteristics of the random matrix, the secret information set to be retrieved is very small, reducing the modification range of the steganography image and improving the steganography image quality and efficiency. This scheme can maintain the steganography image quality with a PSNR of 49.95 dB and steganography of 1.5 bits per pixel and can ensure that the steganography efficiency is improved by reducing the steganography set. In order to adapt to different steganography requirements and improve the steganography ability of the steganography schemes, this paper also proposes an adaptive large-capacity information-hiding scheme based on the random matrix. In this scheme, a method of expanding the random matrix is proposed, which can generate a corresponding random matrix according to different steganography capacity requirements to achieve the corresponding secret-information steganography. Two schemes are demonstrated through simulation experiments as well as an analysis of the steganography efficiency, steganography image quality, and steganography capacity and security. The experimental results show that the latter two schemes are better than the first two in terms of steganography capacity and steganography image quality.

## 1. Introduction

In recent years, people have continued to pursue high-pixel pictures, and the storage space occupied by pictures is also increasing. In the network environment, pictures are an important way of communication, such as in various instant chat systems and cloud photo album systems. There are countless pictures transmitted and uploaded every day. Now, some handheld devices can capture 7296 × 5472 high-pixel images. This size can store more than 500 pictures. If such a large picture is transmitted directly through the network, the other party cannot receive the message immediately, which will affect the user experience and consume a large amount of limited network bandwidth; if such a large image is directly stored on the server, it will bring huge pressure onto the server and consume a lot of storage costs. Because of the huge amount of data, the original image is difficult to transmit, store, and process, so image compression is very important. Some data in the image is redundant, which is the main basis of compressed images.

With the development of the image and video industry, image coding technology has also been accompanied by continuous progress and change [1]. Image compression coding can be divided into two main processes: encoding and decoding. For the coding process, most coding methods first map the image in order to remove the correlation between the pixels and make the data easier to be coded. After that, the image goes through a lossy quantization process, which uses different quantization values to approximate the original data in segments and can be subdivided into uniform quantization and non-uniform quantization according to whether the quantization interval is equally divided so as to reduce the number of coded bits of each codeword. Finally, entropy coding is carried out to convert the quantized data into a binary code stream for better storage and transmission. This restores the binary code stream to the data and completes the image reconstruction according to the decoded data. Image quality is evaluated by measuring the objective index under the bit consumption of the same unit pixel. In recent years, the coding algorithm has not only made a breakthrough in principle, but its application is also developing with display technology. For example, the Guangzhou treasure naked-eye 3D was specially selected at the 2021 ICIF; the world’s first naked-eye 3D giant panda live-action blockbuster also appeared in Chengdu Taikooli in November of the same year.

**Figure 1:**
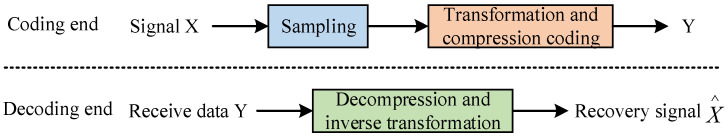
Block Diagram of Image Decoding.

Light-field imaging has become a technology that can obtain more abundant visual information. Traditional photography captures the two-dimensional projection of light in the scene [2]. In contrast, light-field imaging collects light brightness in all directions through angle domain integration and decomposes angle information lost in traditional photography. On the one hand, this high-dimensional visual data representation provides a powerful capability for scene understanding and improves depth perception, refocusing after capture, video stability, and other performance of traditional computer vision. On the other hand, the high dimension of the optical field also brings new challenges to data capture and data compression. If we want to further apply light-field imaging technology to production and life, we need to solve the problem of image data coding. In traditional signal processing theory, signal processing generally needs to go through four processes: sampling digitization, compression coding, storage and transmission, and recovery and reconstruction, as shown in Figure 1. Nowadays, people have higher and higher requirements for images, videos, and other data. Such sampling will generate massive data, which brings not only great pressure to signal storage and transmission but also great challenges to hardware acquisition equipment [3]. In addition, after we transform all the sampling values, we only retain a small proportion of the large coefficients and discard most of the remaining small coefficients when encoding and transmitting, which is undoubtedly a waste of computing resources.

Image hiding technology is an information-hiding technology based on the digital image. It is a technology that makes use of the redundancy of digital images and the insensitivity of human vision to hide some specific information in the image carrier data. In image hiding, the hiding of specific information does not affect the essential characteristics or use value of the image carrier signal. Image hiding is able to transmit some specific information through the channel of transmitting images. The receiving end separates the hidden information through a specific decoder. The principle of information hiding in the space–time domain is easy to understand, and the operation process is simple and has certain advantages. Reference [4] first proposed two methods based on modifying the LSB plane of the carrier. Reference [5] trades off the main evaluation indicators of image hiding technology between robustness and invisibility and then proposes a generalized LSB algorithm. References [6,7,8] first scramble the image and then hide different amounts of information on the three color components of R, G, and B, thereby improving the standard LSB algorithm.

Transmitting through public channels provides a hidden and secure communication method for both parties. Information hiding is generally carried by media such as video, audio, text, and image, which are common on the Internet. Because the number of images in the public network is larger than that in other carriers, and the computer program calculation is relatively simple when information hiding is carried out, using images as carriers has natural advantages and better application and research value. In the field of image-based information hiding, the main evaluation indexes related to the effect of information hiding include the quality of the steganographic image and the steganographic capacity. However, these two indicators are often opposed to each other. The secret information hidden in the original image is a kind of “noise” for the image, and the more data that is hidden, the more “noise” the steganographic image contains and the worse the image quality. Therefore, on the one hand, it is necessary to increase the capacity of the secret information in the picture as much as possible to ensure that the secret information can convey enough information. On the other hand, the secret information should not be detected by steganalysis technology used to determine the existence of secret information or deleted or disturbed by malicious attackers.

Therefore, how to make the steganographic capacity and the quality of steganographic images more satisfactory has become the focus of researchers.

## 2. Related Works

Compared with spatiotemporal image hiding, transform domain image hiding can control the embedding of information to be hidden by applying the perception ability of the human visual system to different time–frequency features, that is, to ensure its invisibility and obtain better image quality. There is no significant difference in energy distribution between the transformation domain and space domain.

The coefficient of the high-frequency part representing the abrupt component of the signal is relatively small, which means that some information can be hidden in the high-frequency part. References [9,10] proposed a hiding algorithm based on 3D discrete cosine transform. References [11,12] use carrier images transformed by wavelet packets to hide information. References [13,14] proposed an image hiding algorithm in the discrete Fourier transform domain. References [15,16] proposed a DCT algorithm to change the pixel length to achieve image hiding. Using the basis of research and analysis on the integer wavelet sequence, reference [17] proposed a method based on information sharing, which improved the information embedding amount of the image hiding algorithm after optimization. After that, the energy aggregation and matrix of the shearlet transform were analyzed. Given the good stability of the singular values, an improved image hiding algorithm is proposed. References [18,19,20,21] proposed a complex valued encrypted image hiding technology based on carrier image DCT, which realized the hiding and extraction of the image information in the carrier images. Therefore, after extracting the data hidden in the picture, the original image can be restored without loss. However, this steganography method is relatively fragile and needs to be transmitted through a safe and reliable channel to ensure that it will not be lost in the transmission process. References [22,23,24,25] proposed that irreversible information hiding technology uses a large number of social pictures on the Internet as carriers and shelters to transmit secret information and pays more attention to the transmission of secret information. Because the transmission is conveyed through unreliable channels, the transmission itself may affect the steganography image, so some robustness of the confidential information is required.

To sum up, this paper designs a new reference matrix random matrix according to the Sudoku matrix, other reference matrices, and the properties of a chaotic system and embeds secret information into the original image through the guidance of the reference matrix so that the image with the secret information has better invisibility and higher security.

## 3. Random Matrix Model and Image Coding Hiding

### 3.1. Singular Value Decomposition of Random Matrices

Singular value decomposition (hereinafter referred to as SVD) is an algorithm widely used in the field of machine learning. It is used to calculate the pseudo-inverse of a matrix so as to solve linear least squares and least squares problems. It can be used not only for feature decomposition in a dimension reduction algorithm but also in a recommendation system, natural language processing, and other fields. SVD has important applications in many fields, such as digital image analysis and processing, background modeling, information security, etc. Singular value decomposition is a very basic operation algorithm in linear algebra matrix calculation. The main idea is to divide the original data matrix into three matrices, of which U and V are orthogonal matrices. Its main steps are divided into two steps:

(1) $$A=U_{1}\times B^{*}V_{1}^{T}$$

(2) $$B=U_{2}\times A\times V_{2}^{T}$$

Among them, each element of the matrix corresponds to an eigenvalue of matrix *A*, and they are expressed in descending order; then, each column in matrix *U* represents the right singular vector of matrix *A*, and each column represents the left singular vector of matrix *A*. Therefore, to sum up, the singular value decomposition of an ordinary matrix *A* can be obtained as follows:

(3) $$A=\left(U_{1}\times U_{2}\right)\times \Lambda \times \left(V_{2}^{T}\times V_{1}^{T}\right)$$

The decomposed 3 matrix structures are as follows:

(4) $$U=\left[u_{1}u_{2}\cdots u_{p}\right],V=\left[v_{1}v_{2}\cdots v_{p}\right],\;\Sigma =\left[\begin{array}{cccc} \sigma_{1} & 0 & 0 & \cdots \\ 0 & \sigma_{2} & 0 & \cdots \\ 0 & 0 & \sigma_{3} & \cdots \\ 0 & 0 & 0 & \ddots \end{array}\right]$$

Many algorithms have been proposed to achieve the singular value decomposition of matrices, but in the optimization problem of kernel norm minimization, it is generally only required to solve singular values greater than a certain threshold and their corresponding singular vectors. For this reason, it is very important to discover a simple and computationally efficient singular value decomposition algorithm.

Ultimately, the matrix rank minimization problem can be reduced to kernel norm minimization or weighted kernel norm minimization. The low-rank matrix recovery problem has been mentioned in many coding engineering and applied science fields, and rank minimization techniques have attracted extensive attention. Rank minimization is the key to low-rank solutions and is required in many mathematical models in computer vision and machine learning. It is quite difficult for us to solve such a problem and obtain complete and correct information from a bunch of information with wrong information or tainted information. Usually, such problems have certain sparse and low-rank properties. After the unremitting efforts of many researchers and repeated experiments, they finally concluded that under certain conditions, in order to transform the original problem into an easy-to-solve convex optimization problem, the researchers found that the corresponding relaxation transformation can be performed. In addition, this class of problems has a nice separable structure, which is a convex optimization problem. When dealing with large-scale data matrices, the kernel norm of matrix minimization is usually solved as follows:

(5) $$\underset{W\in R^{m\times n}}{\min}\mathrm{rank}\left(X\right)\;s.t.\;A(X)=b$$

The image encoding model uses the nuclear norm to relax the problem, which is the convex optimization of the rank function, so we use the nuclear norm to replace the rank function and transform to solve the following convex optimization problem:

(6) $$\underset{W\in R^{m\times n}}{\min}\|X{\|}_{*}\;s.t.\;A(X)=b$$

In our practical application, the hidden problem of image coding needs to be considered, so the rank minimization problem also has various external interference noises, which is expressed as follows:

(7) $$\underset{W\in R^{m\times n}}{\min}\|X{\|}_{*}+\frac{\gamma }{2}\|A(X)-b{\|}_{2}^{2}$$

(8) $$\underset{W\in R^{m\times n}}{\min}\tau ||X|{|}_{*}+\frac{1}{2}||X|{|}_{F}^{2}\;s.t.\;A(X)=b$$

(9) $$\underset{i}{\max}||U^{*}e_{i}|{|}^{2}\leq \frac{\mu \gamma }{n_{1}},\underset{i}{\max}||V^{*}e_{i}|{|}^{2}\leq \frac{\mu \gamma }{n_{2}},UV^{*}{}_{\infty }\leq \sqrt{\frac{\mu \gamma }{n_{1}n_{2}}}.$$

### 3.2. Image Coding Hiding

Today’s image transmission systems usually use the method of parameter sensor perception and image sensor combined with image transmission technology to complete the measurement of aircraft parameters. Compared with the image information, the image transmission data has a very small amount of data, and the use of a channel in communication with the ground leads to a waste of channels.

**Figure 2:**
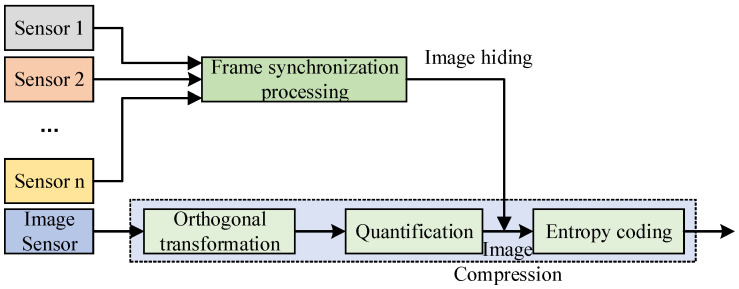
Multichannel image information hybrid transmission scheme.

With the rapid increase of image transmission information and the increasing tension of channel resources, it is believed that the low-frequency and important image transmission information obtained with parameter sensors and the broadband image information obtained with image sensors are hybrid transmissions based on image hiding. In view of the separation of multi-sensor image transmission data at the receiving end and the large amount of image data information, the image transmission data obtained using a multi-sensor before mixed transmission is subject to frame synchronization preprocessing and image compression coding. The specific multichannel image transmission data mixed-transmission design scheme is shown in Figure 2.

**Figure 3:**
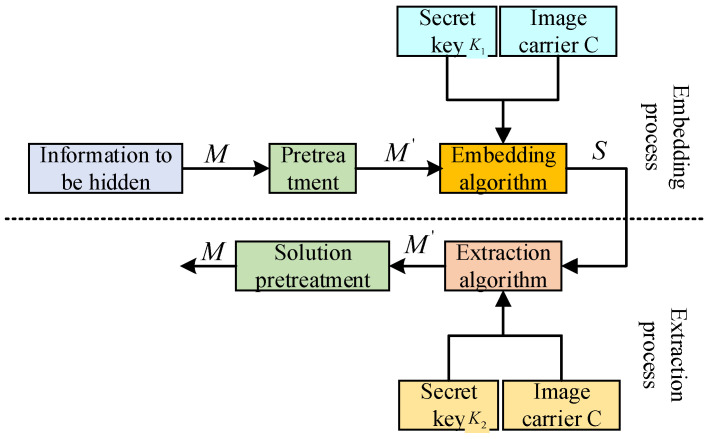
Frame model of image hiding.

On this basis, the generalized model of image hiding is shown in Figure 3. The general model of image hiding includes the embedding process, the transmission process, and the extraction process of the information to be hidden. The embedding process of image hiding should first preprocess the information *M* to be hidden (such as encryption or spread spectrum, etc.) to obtain the processed message *M*. Then, using the key $K_{1}$ and hiding it into the image carrier C through a specific embedding algorithm, the hidden information *S* is obtained. During the transmission process, the information *S* may be illegally intercepted by a third party and re-sent after malicious processing. The receiver uses the extraction algorithm and the key $K_{2}$ corresponding to the embedding algorithm to extract the hidden information from the hidden information *S* to obtain the message $M^{\prime }$ and then obtain the original message *M* through de-preprocessing. In general, private key information is used to achieve hiding, $K_{1}=K_{2}$.

In the extraction process, the hiding technology that does not require the participation of the original image C is called blind information hiding technology; otherwise, it is called non-blind information hiding technology. In the actual experimental design, because the non-blind information hiding technology requires the original carrier C, it is more likely to attract the attention of others and waste channel resources. Within the in-depth research on image hiding technology, most of the focus is on blind information-hiding technology. The general model of image hiding is briefly introduced here, and the detailed implementation process will be further introduced in Section 4. Table 1 lists two alternative generalized Barker code sequences, in which the codewords are represented in octal format.

When j=0, $R(0)=\sum_{i=1}^{1}x_{i}^{2}=1+1+1+1+1+1+1=7$;

When j=1, $R(1)=\sum_{i=1}^{t}x_{i}x_{i+1}=-1-1+1-1+1+1=0$;

When j=2, $R(2)=\sum_{i=1}^{5}x_{i}x_{i+2}=1-1-1-1+1=-1$;

When j=3, $R(3)=\sum_{i=1}^{i}x_{i}x_{i-3}=1+1-1-1=0$;

When j=4, $R(4)=\sum_{i=1}^{3}x_{i}x_{i+4}=-1+1-1=-1$;

When j=5, $R(5)=\sum_{i=1}^{2}x_{i}x_{t+s\;}=-1+1=0$;

When j=6, $R(6)=\sum_{i=1}x_{i}x_{i+5}=-1$.

Table 2 is the specific image coding hidden data framing format, in which the first frame data only includes the system parameters and the number of sampling channels, and then each frame of data is packaged and encoded according to the second frame data format, 192 bit, and one frame of data includes 8-channel 12 bit analog quantity and 1-channel 8 bit digital quantity.

**Figure 4:**
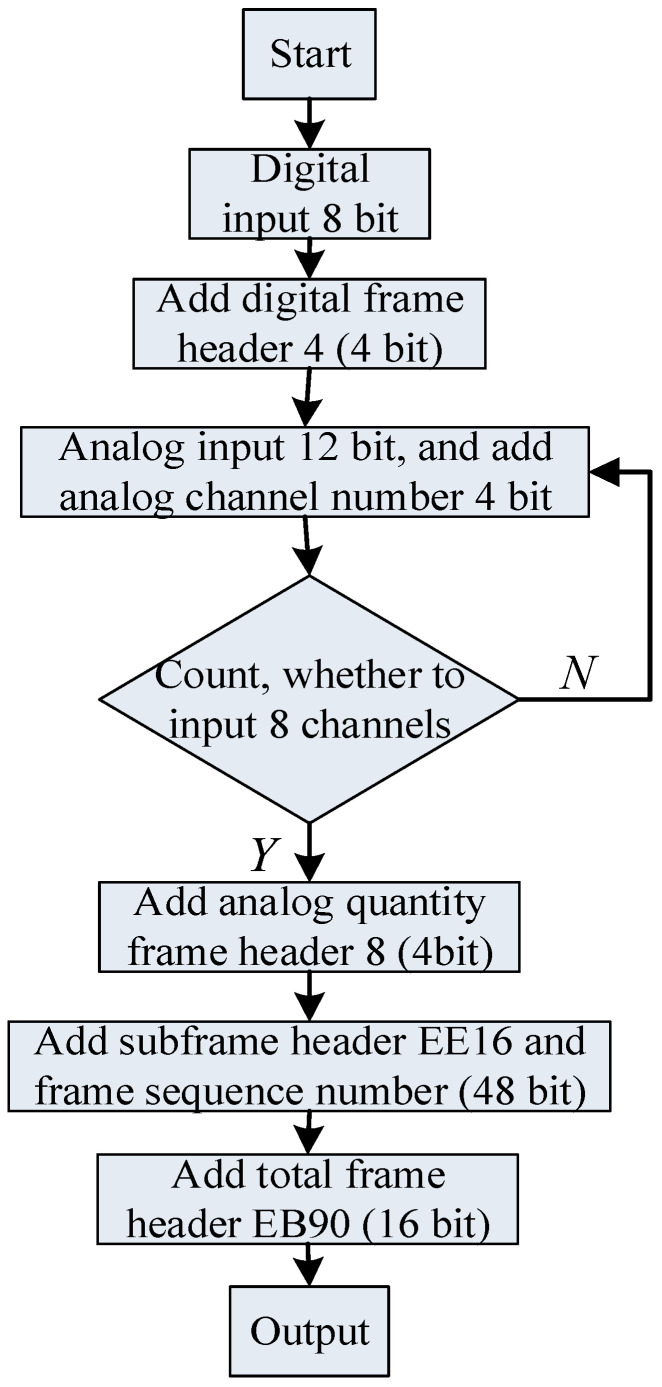
Image coding signal preprocessing.

According to the data framing format in Table 2, this paper uses multiple sets of Barker codes and mixed framing methods on the Matlab 2016 A to realize the preprocessing of image-coded signals. The specific coding design and implementation process are shown in Figure 4.

**Figure 5:**
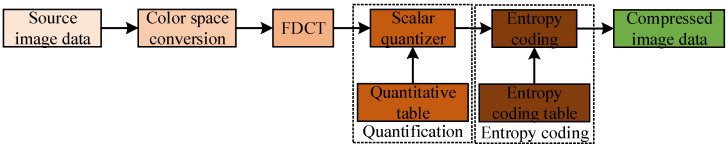
Encoding block diagram based on JPEG image compression.

At present, three operations of domain transformation, quantization, and encoding are included in all image coding concealment schemes, but the corresponding methods of the three operations adopted by the different coding schemes are different. Figure 5 shows the key processing steps and core content of DCT-based coding.

It is calculated as follows:

(10) $$F_{\mu_{j}-0,R_{j}}\left(\delta^{\prime }\right)=\frac{15}{8\pi \sqrt{7^{3}\left|\sigma_{j}\right|}}\int_{z_{i}^{\prime }}^{z_{2}^{’}}\left(1-\frac{1}{7}\varphi^{’T}\sigma_{j}^{-1}\varphi^{\prime }\right)dz^{\prime }=\frac{15}{8\pi \sqrt{7^{3}\left|\sigma_{j}\right|}}\cdot \frac{4\Delta^{\frac{3}{2}}}{21\sigma_{f}^{2}}=\frac{15}{8\pi \sqrt{7^{3}\left|\sigma_{j}\right|}}\cdot \frac{4|\sigma {|}^{\frac{1}{2}}{\left[7\left|R_{j}\right|-\delta^{\prime \tau }\left|R_{j}\right|R_{j}^{-1}\delta^{\prime }\right]}^{\frac{3}{2}}}{21{\left|R_{j}\right|}^{2}}=\frac{5}{14\pi \sqrt{\left|R_{j}\right|}}{\left(1-\frac{1}{7}\delta^{\prime T}R_{j}^{-1}\delta^{\prime }\right)}^{\frac{3}{2}}$$

**Figure 6:**
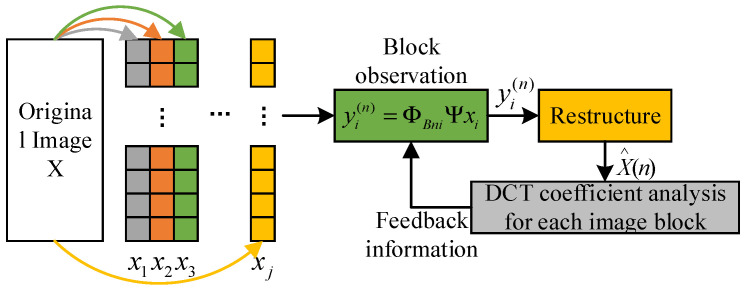
Image encoding and hiding methods.

In simple terms, the image coding concealment is an 8bit grayscale image; however, the image coding concealment has completely different characteristics and uses from texture images. First of all, in terms of the meaning of the pixel value, 0 represents the farthest from the human eye, and 255 represents the closest to the human eye, while the pixel value in the texture image represents the intensity or gray level of the light-sensing point at this point, which is the contrast between the image coding concealment and the texture image. Secondly, from the perspective of gray-level distribution, the gray-level distribution of the image coding concealment is obviously different from that of the texture image. Because depth information only represents the distance between the camera and the object, there is no obvious texture in the image coding concealment. The areas contained by an object have very similar gray levels, but there are obvious gray-level differences at the edges of the object. Finally, the image coding concealment and texture image are also very different in time continuity. The existing depth estimation algorithms and depth acquisition devices are not very accurate, which makes the inter-frame continuity of the image coding concealment significantly lower than that of texture video frames. The image display mode is shown in Figure 6.

## 4. Methods

**Figure 7:**
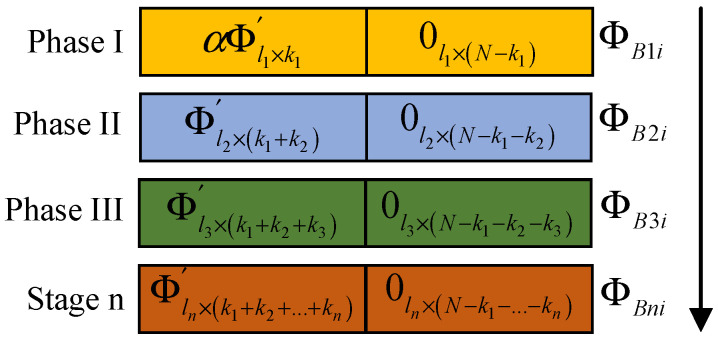
Schematic diagram of the image encoding end generating the observation matrix in stages.

**Figure 8:**
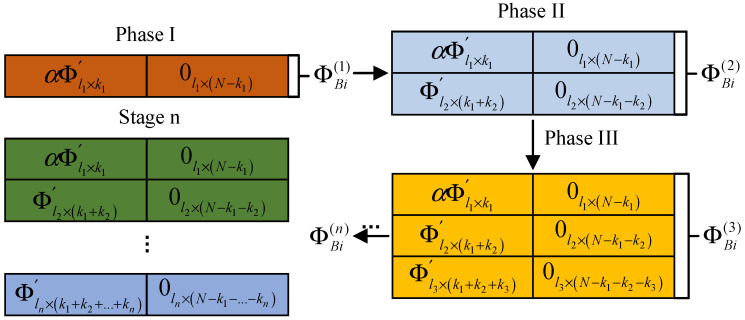
Image coding hidden observation matrix.

The observation matrix used in this scheme is updated in stages. In the first stage, each block uses the same observation matrix to observe several important low-frequency coefficients. In the subsequent stages, block-wise DCT coefficient analysis is carried out on the recovered image from the previous stage, and the analysis results are fed back to the encoding side as a priori information to guide the updating and optimization of the observation matrix. As shown in Figure 7, the encoding side generates the observation matrix in stages. The observation matrix used in the second stage is updated according to the feedback information from the decoding terminal in the first stage. The decoding terminal feeds back the position of the large coefficient that is greater than the threshold value. The position of the observation matrix corresponding to the large coefficient is a random matrix, and other smaller coefficients are observed corresponding to the zero matrix. In this way, the observation reconstruction in the next stage is repeated, as shown in Figure 8.

### 4.1. Threshold Selection

**Figure 9:**
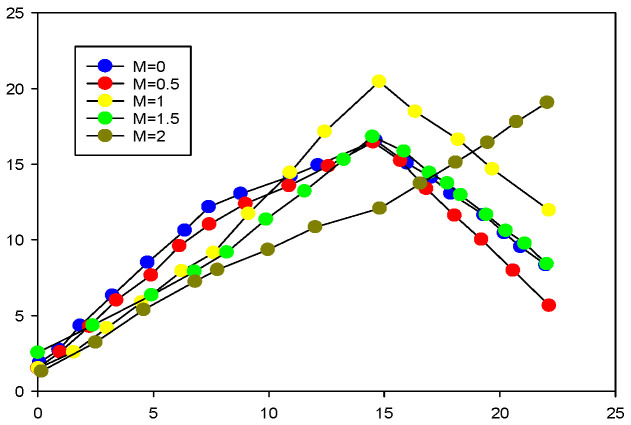
Image coding hidden threshold distribution selection.

In this scheme, the distribution characteristics of the image in the DCT domain are used for encoding and hiding, the DCT coefficients of the image are analyzed at the decoding end, the threshold is set, and the coefficients greater than the threshold are emphatically observed. In this scheme, we determine appropriate thresholds for different images through several groups of experiments, and we use the same threshold for all blocks in the image. The performance comparison of four different images when different thresholds are selected is shown in Figure 9. We can see that when certain threshold values are selected and the number of observed values increases, the PSNR performance decreases. This is due to the unreasonable selection of threshold values. For a diamond with more textures, the threshold value is 0.5.

Suppose that the pixel pair extracted from the original image is (4, 1), and the binary secret data is (001) 2. The corresponding random matrix *M* is generated by the initial secret key *k* in Section 3.1, and the element *M* (4, 1) of the pixel pair corresponding to the reference matrix *M* (4, 1) is found according to the extracted pixel pair (4, 1). As shown in Table 3, the element that *M* (4, 1) corresponds to is 4. Because the decimal system corresponding to the secret information is (1), by searching the search set corresponding to *M* (4, 1), it can be found that the element corresponding to the secret information is *M* (3, 1), Therefore, the extracted pixel pair (4, 1) is adjusted to (3, 1) to complete the steganography of secret data (001) 2. In the secret-information-extraction stage, by extracting pixel pairs (3, 1) at the same position of the steganography image and generating the same random matrix *M* according to the user’s initial secret key, according to the extracted pixel pairs, the corresponding pixel value *M* (3, 1) = (1) 9 in the random matrix; converting it into binary data, the extracted secret data is (001) 2.

Table 4 shows the differences in information-hiding schemes based on a Sudoku matrix when hiding secret information of the same size. The steganography image quality generated by this scheme is higher, and the average PSNR is 4.98 dB higher than that of the Sudoku matrix scheme [26]. Compared with the scheme based on a turtle shell matrix, under the same steganography capacity EC, the PSNR of this scheme is also improved by 0.55 dB because of its lower security. In the case of an information-hiding algorithm, anyone can extract steganographic data using steganography, and the ability to protect secret information is poor.

The search scope of the image-encoding hiding is among the three turtle shell hexagons; that is, hiding 8bit secret information needs to be searched in 16–24 elements. The secret information is encoded first followed by steganography, which creates additional computational overhead. In this scheme, a search range is a set containing nine elements, and because of the security of the reference matrix, there is no need for additional encryption or a calculation to scramble the steganography order, and the calculation cost is low, as shown in Table 5.

### 4.2. Secret Information Extraction

In order to explain the steganography stage and extraction stage of this extension scheme more clearly [27], the information steganography process of this scheme is demonstrated in Table 6.

In the secret-information-extraction stage, by extracting pixel pairs (3, 3) at the same position in the steganographic image and generating the same random matrix *M* according to the user’s initial secret key, the corresponding pixel values in the random matrix according to the extracted pixel pairs are converted into binary data, the extracted secret data (1011) 2. In this example, according to the generated extended random matrix, the scheme can hide 2bit secret data in each pixel value.

## 5. Case Study

In order to prove that the steganography image quality of this scheme is good, this scheme has undergone further experiments. Under the condition that the steganography parameter N = 16, that is, the steganography capacity EC = 2.0 bpp, the PSNR of the corresponding steganography image is shown in Table 7. The classical least significant bit (LSB) scheme is 45.32 dB. It can be found that our scheme provides higher steganography image quality under the same steganography capacity.

To further illustrate the adaptability of the scheme to steganography requirements, Table 8 shows the changes in steganography capacity EC and steganography image quality PSNR under different steganography parameters and ensures the concealment and security of the steganographic images and secret information.

**Figure 10:**
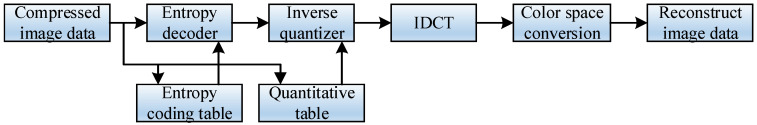
Image coding hidden block diagram.

**Figure 11:**
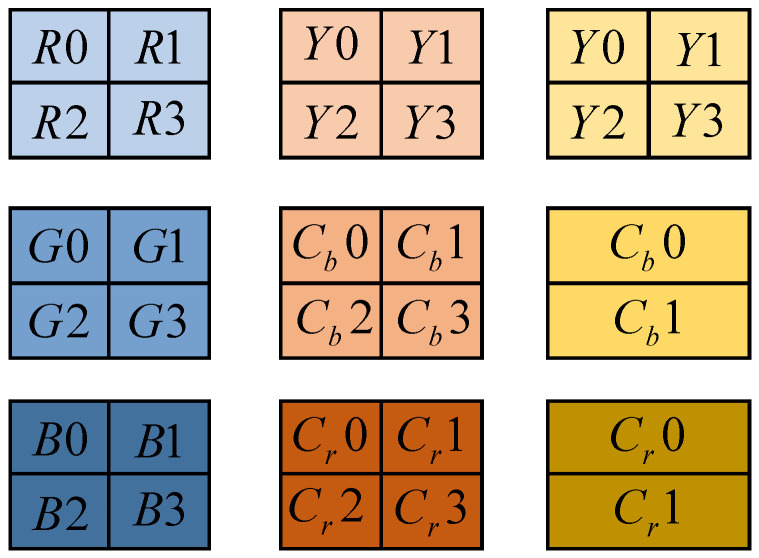
Image coding hidden matrix relationship.

As can be seen from Figure 10 and Figure 11, even with the same original image and the same secret information, the steganographic results generated by the different secret keys are very different, thus verifying the security of this scheme.

For the same transform coefficient, the values of the selected luminance quantization table and color difference quantization table are also different, and the reference value is given in the standard JPEG compression coding, as shown in Table 9 and Table 10.

**Figure 12:**
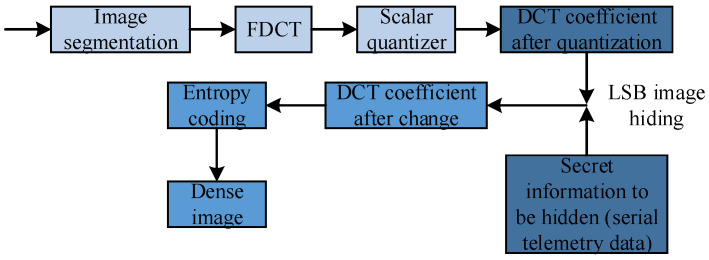
The final optimization model of image coding hiding.

As can be seen from the above table, the quantization table is also an 8 × 8 data matrix, which corresponds to the 64 transform coefficients in the sub-block obtained with DCT. In the quantization table, each element value is an integer, and the value range is 1~255. This represents the quantization step size of the transform coefficients obtained at the corresponding position. The luminance quantization table given in Table 9 is the quantization matrix obtained according to the psychovisual weighting function; the color quantization table given in Table 10 is the quantization matrix obtained according to the psychovisual weighting function, as shown in Figure 12.

**Figure 13:**
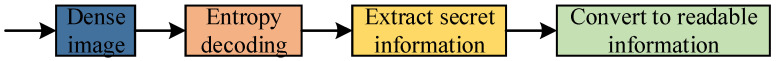
Information-extraction module hidden by image coding.

Among them, the secret information is further compressed through entropy coding and other operations, and then the encrypted image is obtained in JPEG format. The extraction of the secret information is just the opposite of the embedding process in Figure 13. First, the corresponding entropy decoding is performed on the encrypted image in JPEG format, and the quantized DCT coefficient matrix is obtained with the secret information hidden. Then, according to the corresponding extraction algorithm, the secret information is extracted from the DCT coefficient matrix.

Table 11 gives different (1, *n*, *k*) corresponding matrix codes to optimize the performance of LSB image hiding.

To sum up, in this section, aiming to separate the multi-sensor data at the receiving end, the frame synchronization method of coherent insertion of Barker code is adopted to realize the preprocessing of telemetry data. In the Section 2, the coding design process is completed. Next, the coding is completed using the measured signal based on the Matlab software platform to complete the simulation verification. The coding results are obtained, and the effective separation of multichannel data is realized. The results are analyzed for performance.

## 6. Conclusions

Determining the optimal observation and quantization coding scheme of a depth image based on compressed sensing is an important path for image coding concealment. In this scheme, the encoding side observes the depth image in pairs with variable density in the Fourier domain and then performs dead-zone quantization and arithmetic coding on the obtained observations. For the sparsity of the image in the pixel domain, TV (Total Variation) constraints are added to ensure the correct restoration of the edge of the depth image. Through experiments to find the optimal relationship between the observation rate and the quantization level, the rate-distortion performance of the algorithm is optimized. The experimental results show that this scheme can maintain the edge information of the depth image better than the traditional JPEG and JPEG2000 methods and guide the visual effect of the synthesized image. In addition, because the corresponding reference matrix can only be generated through the initial secret key and the corresponding chaotic system, the embedded secret information is similar to the traditional encryption scheme without the secret key, and it is difficult to obtain effective information from it, which is easily mistaken for the noise of the picture. The picture quality is higher, the concealment of the picture with the secret information is improved, and the steganography efficiency is also improved.

## Figures and Tables

**Table 1:** List of optional parameter sequences.

Length	Sequence Type A	Sequence Type B	Length	Sequence Type A	Sequence Type B
4	10	4	13	16,540	17,456
7	130	130	14	34,640	37,145
8	270	-	15	73,120	76,326
9	560	-	16	165,620	167,026
10	1560	-	17	363,420	317,522
11	2670	2670	18	746,500	764,563
12	6540	-	19	1,746,240	1,707,355

**Table 2:** Image coding hidden data framing format.

First Frame	Total Frame Header	Subframe Header	Frame Sequence Number	Sampling Frequency	Number of Channels			
Second frame	Data framing	Subframe header 16 bit	Frame sequence number 32 bit	Analog frame header 4 bit	Analog channel number 4 bit × 8	Analog channel data 12 bit × 8	Digital quantity frame header 4 bit	Digital channel data 8 bit

**Table 3:** Pixel extraction matrix.

	0	1	2	3	4	5	6	7	8	9	…
0	6	1	7	5	2	0	3	4	8	5	…
1	3	4	8	6	1	8	6	1	0	6	…
2	5	2	0	3	4	7	5	2	7	3	…
3	6	1	7	5	2	0	3	4	8	5	…
4	3	4	8	6	1	8	6	1	0	6	…
5	5	2	0	3	4	7	5	2	7	3	…
6	6	1	7	5	2	0	3	4	8	5	…
7	3	4	8	6	15	8	6	1	0	6	…
8	5	2	0	3	4	7	5	2	7	3	…
…	…	…	…	…	…	…	…	…	…	…	…

**Table 4:** Comparison of evaluation metrics in different image coding concealment.

Images	EMD		Based on Sudoku		Based on Turtle Shell Matrix		This Scheme	
	PSNR	EC	PSNR	EC	PSNR	EC	PSNR	EC
Lena	49.42	1.37	44.97	1.5	49.42	1.5	49.95	1.5
Peppers	50.78	1.37	44.67	1.5	49.4	1.5	49.95	1.5
Baboon	50.69	1.37	44.68	1.5	49.36	1.5	49.96	1.5
Boat	50.64	1.37	44.94	1.5	49.4	1.5	49.95	1.5
Man	50.82	1.37	44.96	1.5	49.42	1.5	49.95	1.5
House	50.82	1.37	44.96	1.5	49.42	1.5	49.97	1.5

**Table 5:** Security of image coding matrix.

Security	Search Collection	Additional Expenses
Scrambling-based steganography order	Small	Scramble steganography order
Encrypt secret information	More	Nothing
Encrypt secret information	More	Nothing
Based on reference matrix	Small	Nothing

**Table 6:** Steganographic examples of image coding information.

…	…	…	…	…	…	…	…	…	…	…	…
8	0	1	14	12	6	1	7	3	2	13	…
7	2	13	7	11	15	13	9	12	6	4	…
6	6	4	10	8	0	4	14	11	0	5	…
5	15	5	9	3	2	5	10	8	15	1	…
4	0	1	14	12	6	1	7	3	2	13	…
3	2	13	7	11	15	13	9	12	6	4	…
2	6	4	10	8	0	4	14	11	0	5	…
1	15	5	9	3	2	5	10	8	15	1	…
0	0	1	14	12	6	1	7	3	2	13	…
	0	1	2	3	4	5	6	7	8	9	…

**Table 7:** Comparison of image steganography.

Image	LSB	[15]	[20]	This Scheme
Lena	45.33	43.03	47.62	47.86
Peppers	45.32	42.54	47.62	47.86
Baboon	45.32	41.41	47.59	47.83
Boat	45.32	42.17	47.61	47.85
Pirate	45.32	42.49	47.63	47.87
House	45.32	42.54	47.64	47.88
Airplane	45.32	42.35	47.62	47.86
Average	45.32	42.35	47.62	47.86

**Table 8:** Image steganography parameter results comparison.

Steganographic capacity (EC)	1.5 bpp	2.0 bpp	2.5 bpp	3.0 bpp
Scheme of literature [15]	49.87	46.23	42.86	40.52
Scheme of document [20]	52.33	47.61	44.28	42.57
This scheme	49.96	47.86	44.42	42.63

**Table 9:** Image coding hidden luminance quantization.

21	54	16	74	37	32	56	13
16	56	26	87	56	71	08	25
34	24	64	72	84	85	72	74
11	14	84	47	44	05	225	78
09	52	56	73	43	05	25	75
64	98	24	73	15	68	56	35
56	00	43	37	16	51	35	64
45	86	53	16	72	88	25	99

**Table 10:** Image coding hidden color-difference quantization.

17	18	24	47	99	99	99	99
18	21	26	66	99	99	99	99
24	26	56	99	99	99	99	99
47	66	99	99	99	99	99	99
99	99	99	99	99	99	99	99
99	99	99	99	99	99	99	99
99	99	99	99	99	99	99	99
99	99	99	99	99	99	99	99

**Table 11:** Algorithm performance comparison table.

*k*	*n*	R (%)	E
1	1	100.0	2.0
2	3	66.7	2.7
3	7	42.9	3.4
4	15	26.7	4.3
5	31	16.1	5.2
6	63	9.5	6.1
7	127	5.5	7.1
8	255	3.1	8.0
9	511	1.8	9.0

## Data Availability

The experimental data used to support the findings of this study are available from the corresponding author upon request.
